# Effect of data-driven motion correction for respiratory movement on lesion detectability in PET-CT: a phantom study

**DOI:** 10.1186/s40658-025-00784-x

**Published:** 2025-07-11

**Authors:** Marloes A. de Winter, Robin Gevers, Jules Lavalaye, Jan B. A. Habraken, Matteo Maspero

**Affiliations:** 1 Department of Medical Physics, St Antonius Hospital, Nieuwegein, The Netherlands; 2 Department of Nuclear Medicines, St Antonius Hospital, Nieuwegein, The Netherlands; 3 Department of Radiotherapy, University Medical Center Utrecht, Utrecht, The Netherlands; 4 Computational Imaging Group for MR Diagnostics & Therapy, University Medical Center Utrecht, Utrecht, The Netherlands

**Keywords:** Positron emission tomography, Computed tomography, Motion correction, Artificial intelligence, Respiratory motion correction

## Abstract

**Purpose:**

While data-driven motion correction (DDMC) techniques have proven to enhance the visibility of lesions affected by motion, their impact on overall detectability remains unclear. This study investigates whether DDMC improves lesion detectability in PET-CT using FDG-18F.

**Method:**

A moving platform simulated respiratory motion in a NEMA-IEC body phantom with varying amplitudes (0, 7, 10, 20, 30 mm) and target-to-background ratios (2, 5, 10.5). Scans were reconstructed with and without DDMC, and the spherical targets’ maximal and mean recovery coefficient (RC) and contrast-to-noise ratio (CNR) were measured.

**Results:**

DDMC results in higher RC values in the target spheres. CNR values increase for small, high-motion affected targets but decrease for larger spheres with smaller amplitudes. A sub-analysis shows that DDMC increases the contrast of the sphere along with a 36% increase in background noise.

**Conclusion:**

While DDMC significantly enhances contrast (RC), its impact on detectability (CNR) is less profound due to increased background noise. CNR improves for small targets with high motion amplitude, potentially enhancing the detectability of low-uptake lesions. Given that the increased background noise may reduce detectability for targets unaffected by motion, we suggest that DDMC reconstructions are used best in addition to non-DDMC reconstructions.

**Supplementary Information:**

The online version contains supplementary material available at 10.1186/s40658-025-00784-x.

## Introduction

PET-CT plays a pivotal role in oncology by providing valuable insights into initial cancer staging, assessment of therapeutic response, restaging, and longitudinal surveillance for recurrence [1–3]. However, respiratory motion can hinder accurate lesion identification and assessment in PET-CT, affecting oncology diagnosis [4, 5]. The impact of respiratory motion has become relatively more pronounced due to the improvement in resolution and sensitivity of the newest PET-CT systems [6].

In the last decade, vendors have proposed elastic motion correction (EMC) [7, 8] and artificial intelligence (AI)-based solutions as deviceless motion correction techniques, improving lesion visibility [9, 10]. Retrospective studies on oncological patients compared EMC methods like OncoFreeze (Siemens) and MotionFree (GE Healthcare) with device-based gating to ungated images. Standardized uptake value (SUV) is comparable for EMC and device-based gating, and increase SUV in comparison to ungated images, however the values for contrast-to-noise ratio decrease (CNR) [4].

Data-driven motion correction (DDMC) techniques incorporate previous motion correction techniques with AI, such as OncoFreeze AI (Siemens) [11]. DDMC provides a novel solution for improving image analysis and lesion visualization in the presence of motion. DDMC is based on proprietary image-based anatomical landmark recognition [11, 12], promising fast and robust respiratory motion estimation and correction. Considering the novelty of DDMC, clinical evaluation is crucial for their safe and effective use. Retrospective studies on patient lesions showed increased SUV for DDMC compared to no DDMC [13], but found no additional diagnostic information [14], although CNR was improved [12]. The comparison between device-based gating to DDMC has also been made, and shows small absolute differences between the two, unlikely to be clinically relevant [14]. However, it remains unclear whether DDMC enables the detection of lesions that would otherwise be missed.

This research aims to investigate the impact of DDMC on the detectability of lesions affected by respiratory motion in PET-CT. A phantom study has been designed to characterize the impact of this novel motion correction approach in a controlled environment.

## Material and methods

This study used the NEMA-IEC body phantom to modulate several situations [15], with a comparable setup as used in the EARL IQ measurements [16]. The phantom consists of a body phantom (18 cm length and 9.8 L) and an insert with six hollow spheres with sizes 0.5, 1.2, 2.6, 5.6, 11.5, and 26.5 mL or 10, 13, 17, 22, 28, and 37 mm inside diameter. The body phantom serves as the background and was filled with fluorine-18 (18F) with an activity of about 20 MBq in 9.7 L (2.0 kBq/mL). The spherical targets (0.5–26.5 mL) were filled with three targets-to-background ratios (TBRs) of 2, 5, and 10.5.

**Fig. 1:**
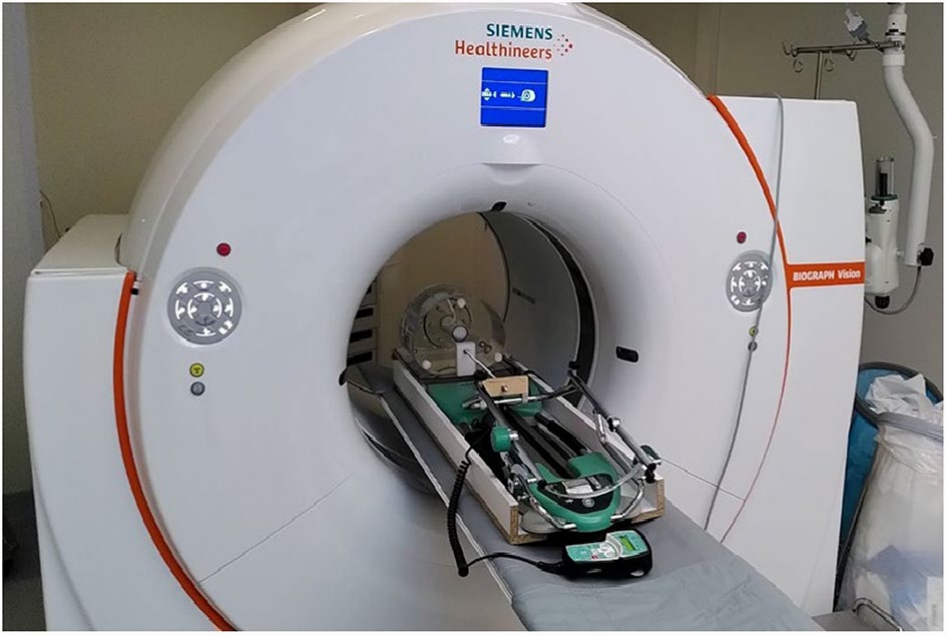
Experimental setup consisting of PET-CT and the adapted Kinetec Spectra enabled motion of the baseplate where the NEMA/IEC body phantom is positioned

Respiratory motion was simulated by adapting a device initially designed for knee rehabilitation (Kinetec Spectra, Medical SOT BV, Losser, The Netherlands). The device was adapted to move the phantom on a 3 mm polymethylmethacrylate baseplate mounted on aluminum rails with amplitudes of 7, 10, 20, and 30 mm at a fixed speed of 0.75 cm/s, mimicking respiratory motion in the lung [17]. The phantom was driven to cycle in the superior-inferior direction with a period of 4 s (Fig. 1). A movie of the setup is included in Supplementary File 1. The scans were performed once for each TBR (2, 5 and 10.5) and amplitude (0, 7, 10, 20 and 30 mm).

### PET-CT protocol

The phantom was scanned on a PET-CT with 4D gating capability (Vision BIOGRAPH 600, Siemens Healthineers, Erlangen, Germany). A 3D-PET/3D-CT protocol was adopted to simulate the most widely used and recommended clinical application protocol for the Vision Biograph 600. PET was 3D attenuation corrected using static and moving 3D-CT, and a PET was reconstructed with corresponding attenuation maps from 3D-CT with and without DDMC. CT had a 512 × 520 matrix size for a 50 cm transaxial FOV. The CT settings are 100 kV, and mA(s) are adjusted according to Siemens’ Care Dose 4D protocol. Following the CT, the 3D-PET data were acquired for 7–8 min with a continuously moving bed position.

PET was acquired with a matrix size of 440 × 440 for 50 cm transaxial FOV with 2 mm slice thickness and reconstructed using a point spread function modeling reconstruction (True X) with time-of-flight correction (TrueX + TOF = ultraHD PET) algorithm (4 subsets, five iterations, and 5 mm FWHM Gaussian post-filter). It was corrected for attenuation using the 3D-CT attenuation map in Hounsfield units (HU). The corrections for the relative scatter and random events were also incorporated into the reconstruction algorithm.

EMC, part of the DDMC algorithm in OncoFreeze, starts with deriving a blurring kernel from the static whole body and HD Chest. Both have the same integral activity, but different brightness and motion blur are solved with mass preservation optical flow [4]. Then, a motion-corrected image estimate is derived using iterative image reconstruction and the deblurring kernel. The AI part in DDMC derives respiratory waveforms from PET raw data using spectral analysis [11, 18], where the total sum of pixel values within a region of interest is proportional to the motion amplitude [11, 19].

### Measurements and parameters

To investigate whether DDMC affects lesion detectability, the volume recovery coefficients (RC) and contrast-to-noise ratio (CNR) are calculated for the target spheres, TBR values, and motion amplitudes. RCs were determined on PET in the volume-of-interest (VOI) with the phantoms in static and dynamic mode using the following formula:

1 $$\text{RC} = \frac{{T}_{observed}/{B}_{mean}}{{T}_{in}/{B}_{in}}$$

where ${T}_{observed}$ is the activity concentration observed in the VOI, ${B}_{mean}$ is the mean activity concentration in the background, ${T}_{in}$ is the activity concentration inserted in the VOI, and ${B}_{in}$ is the inserted activity concentration in the background [15]. For ${T}_{observed}$, the maximum activity (${T}_{max}$) or the mean activity (${T}_{mean}$) in the VOI can be used, resulting in ${RC}_{max}$ and ${RC}_{mean}$, respectively. All activities are in (Bq ⋅ mL^−1^).

To better comprehend the effects of DDMC on movement, ${RC}_{max}$ and ${RC}_{mean}$ are computed. The ${RC}_{max}$, computed using the maximum activity concentration, is more sensitive to image noise than the ${RC}_{mean}$, calculated using the mean activity concentration in the VOI. However, the ${RC}_{mean}$ is more sensitive to the chosen VOI, which is challenging to determine in a moving object.

**Fig. 2:**
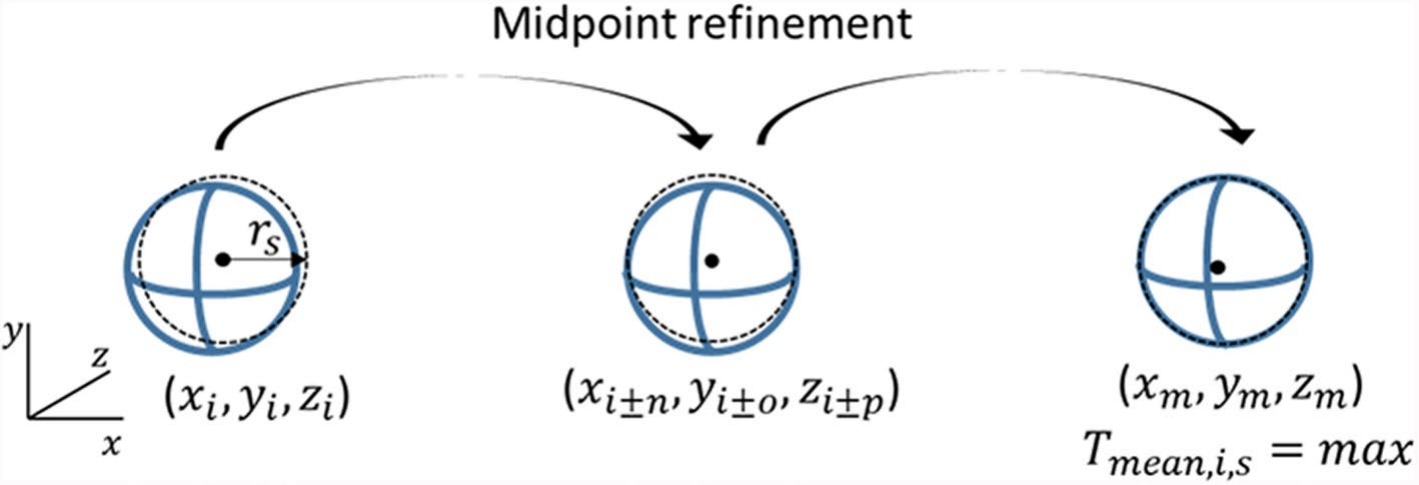
Illustration of the method to find the VOI in the PET CT images: the initial estimate for the target sphere’s midpoint results in a mean activity over the VOI ($${T}_{mean,i,s})$$ is refined by adjusting x, y, and z. This results in multiple candidate midpoints, with the final midpoint selected based on the maximal value for the mean activity ($${T}_{mean,max,s})$$

The target VOI is identified as follows (Fig. 2): First, the target sphere’s midpoint is estimated by clicking on the PET image with the first initial guess (${x}_{i},{ y}_{i}, {z}_{i}$). Given the sphere’s radius (${r}_{s}$)​, the VOI is defined as:$\text{VOI} =( {x}_{i}\pm {r}_{s} ,{y}_{i}\pm {r}_{s} ,{z}_{i}\pm {r}_{s})$. The pixel values within this VOI determine ${T}_{mean,i,s}$ the target sphere (s) with as midpoint the initial guess ($i$), calculated as:

2 $$T_{mean,i,s } = {\text{average}} ( x_{i} \pm r_{s} ,y_{i} \pm r_{s} ,z_{i} \pm r_{s} )$$

To locate the VOI with the highest activity $(T_{mean, max, s} ),$ the initial guess for the midpoint is refined by adjusting $x$ and $y$ by ± 5 pixels and $z$ by ± 3 slices. The smaller range in z-direction accounts for the lower through plane resolution. The refinement results in several midpoints of target spheres (${x}_{i\pm 5}\pm {r}_{s} ,{y}_{i\pm 5}\pm {r}_{s} ,{z}_{i\pm 3}\pm {r}_{s})$. The target sphere’s final midpoint corresponds to the maximal position.

Finally, the background VOI is chosen to be the same ${r}_{s}$ as that of the largest sphere. The position of the background VOI was not optimized but is located at the left posterior corner of the NEMA phantom in the background, which does not include target spheres.

The prepared activity concentration was measured with the Comecer dose calibrator VIK-202. The actual activity in the VOI at the initiation of a scan was calculated by considering the decay of injected activity during the time interval between injection and the initiation of a scan.

The image statistics were evaluated by calculating the contrast-to-noise ratio (CNR). The CNR refers to the ability of the PET to distinguish between various contrasts in an acquired and the inherent noise in the image. The CNR was calculated as

3 $$\text{CNR} = \frac{{T}_{observed}-{B}_{mean}}{{\sigma }_{B}}$$

where the background VOI standard deviation is ${\sigma }_{B}$ [20]. Again, for ${T}_{observed}$, the maximum activity (${T}_{max}$) or the mean activity (${T}_{mean}$) in the VOI can be used to result in ${\text{CNR}}_{max}$ and ${\text{CNR}}_{mean}$, respectively. All activities are in (Bq ⋅ mL^−1^).

The ratio of parameters such as $\text{RC}$ and $\text{CNR}$ is calculated per sphere size (s) and amplitude ($a$) as

4 $$\text{Ratio} (s,a)= \frac{\text{value with DDMC} (s, a)}{\text{value without DDMC} (s, a)}$$

where the parameter values will be averaged over the three TBRs or six sphere sizes for RC and CNR, with “$\text{value with DDMC}$” corresponding to the average RC or CNR values. The parameter value per TBR is not considered if the target sphere or background value is negative. For example, if a CNR with a specific sphere size, amplitude, and TBR is computed and is negative, the ratio parameter will be calculated without that data point.

A statistical analysis is done for the parameters ${RC}_{max}$, ${RC}_{mean}$,${\text{CNR}}_{max}$ and ${\text{CNR}}_{mean}$. The data is paired on amplitude, TBR and sphere size, and statistical tests are done on the parameters with and without DDMC. To check normality of the data, Shapiro Wilk test and quantile–quantile-plot are used, if data is normally distributed a paired t-test is performed, otherwise the Wilcoxon signed rank test is used. The resulting test statistics and p-values are reported per parameter.

The change in background standard deviation (${\sigma }_{B}$) will be averaged over the amplitude settings.

## Results

**Fig. 3:**
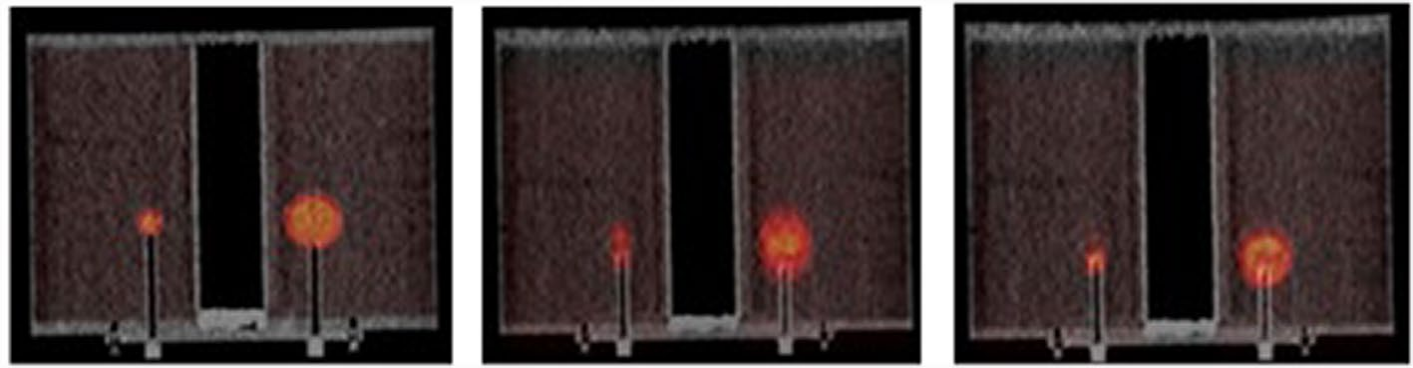
Fused 3D PET/CT image, for a static image (left), motion amplitude 30 mm (middle and right) without and with DDMC, TBR 5

**Fig. 4:**
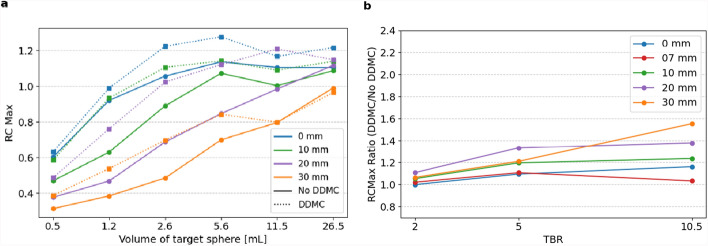
${RC}_{max}$ **a** with and without DDMC, for amplitudes of 0, 10, 20, and 30 mm for TBR 5, with the target spheres on the x-axis. The results with amplitude of 7 mm are omitted from this figure to increase readability. Ratio of ${\text{RC}}_{max}$ **b** with and without DDMC, for amplitudes of 0, 7, 10, 20, and 30 mm averaged for sphere sizes, with the TBR values on the x-axis

The motion correction algorithm reduced image blur originating from movement (Fig. 3). DDMC increasingly affects ${\text{RC}}_{max}$, especially with decreasing sphere size, increasing TBR and increasing amplitude (Fig. 4).

**Fig. 5:**
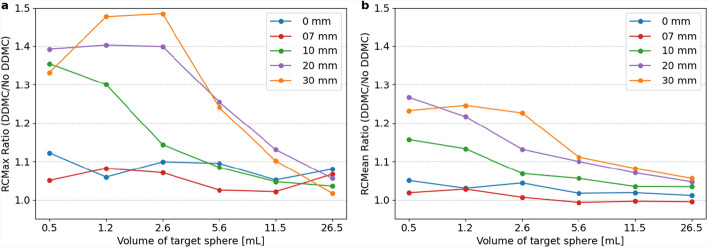
Ratio of ${\text{RC}}_{max}$ (**a**) and ${\text{RC}}_{mean}$ (**b**) with and without DDMC, for amplitudes of 0, 7, 10, 20, and 30 mm averaged for TBR 2, 5, and 10.5, with the target spheres on the x-axis from big to small

Figure 5 shows the ratio of ${\text{RC}}_{max}$ and ${\text{RC}}_{mean}$, averaged over the TBRs with and without DDMC. DDMC increased the average ${\text{RC}}_{max}$ value, with a greater effect for higher amplitude, higher TBR and smaller sphere size. ${\text{RC}}_{max}$ and ${\text{RC}}_{mean}$ for a sphere with a volume of 1.2 mL and amplitude of 20 mm increased by 40.3% and 21.7%, respectively. For the sphere with a volume of 11.5 mL and amplitude of 7 mm, values changed by 1.9% and − 0.5%, respectively. A Wilcoxon signed-rank test revealed a statistically significant difference in ${\text{RC}}_{max}$(W = 112, *p* = 6.82∙10^–15^, n = 90) and ${\text{RC}}_{mean}$ (W = 391, *p* = 2.64∙10^–11^, n = 90).

**Fig. 6:**
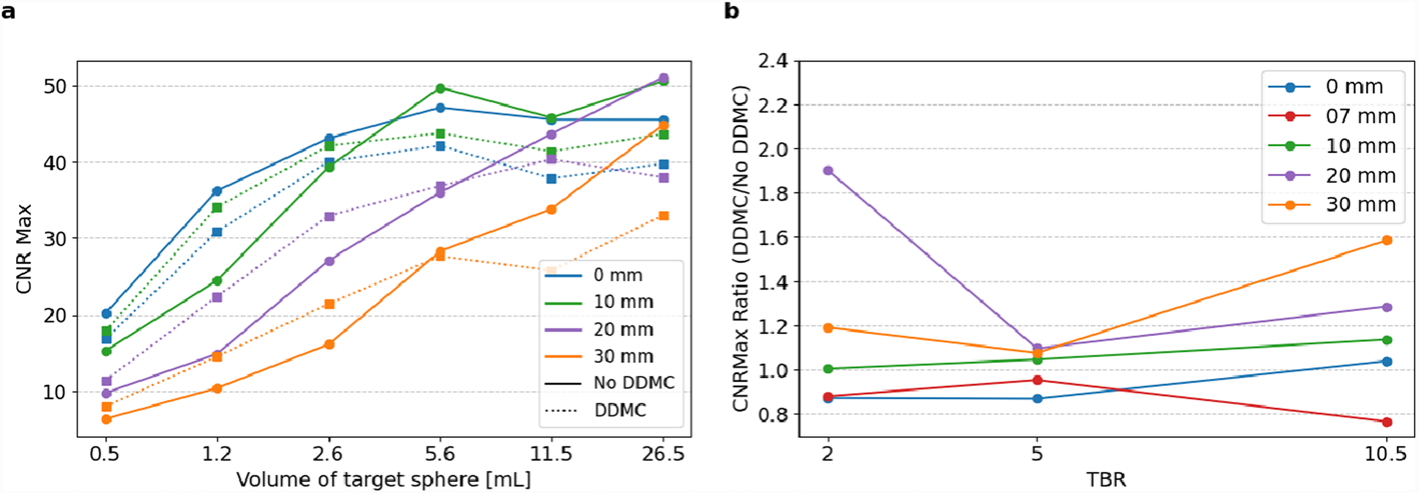
${\text{CNR}}_{max}$ **a** for amplitudes of 0, 10, 20, and 30 mm at TBR 5, with the target spheres on the x-axis from big to small. The results with amplitude of 7 mm are omitted from this figure to increase readability. The ratio of ${\text{CNR}}_{max}$ **b** with and without DDMC, for amplitudes of 0, 7, 10, 20, and 30 mm averaged for sphere sizes, with the TBR values on the x-axis

**Fig. 7:**
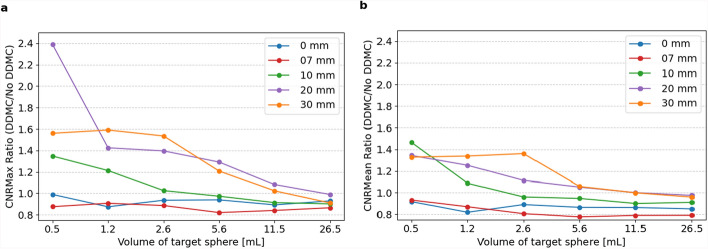
Ratio of ${\text{CNR}}_{max}$ (**a**) and ${\text{CNR}}_{mean}$ (**b**) with and without DDMC, for amplitudes of 0, 7, 10, 20, and 30 mm averaged for TBR 2, 5, and 10.5, with the target spheres on the x-axis from big to small

Figure 6 shows DDMC results on ${CNR}_{max}$. The ${CNR}_{max}$ and ${CNR}_{mean}$ averaged results over all TBRs are visible in Fig. 7 per amplitude and sphere size. $\text{CNR}$ values below zero are not considered when calculating the averaged ratio; this was the case for Fig. 7a, ${\text{CNR}}_{\text{max}}$ with an amplitude of 30 mm and a volume of the target sphere of 0.5 mL. In Fig. 7b, ${\text{CNR}}_{\text{mean}}$ with an amplitude of 20 mm, a volume of target sphere 0.5 mL with an amplitude of 30 mm, and a volume of target spheres 1.2 and 0.5 mL, values were not considered in the averaged ${\text{CNR}}_{\text{mean}}$.

For the TBR averaged $CNR$ values, spheres with high motion amplitude (20 mm) and small sphere size (2.6 mL), the $CNR$ value increased with DDMC, with 39.2% and 11.6% for ${CNR}_{max}$ and ${CNR}_{mean}$, respectively. However, for smaller motion amplitude (10 mm) and bigger sphere size (11.5 mL), $CNR$ values decreased by 8.5% and 10%, respectively. A Wilcoxon signed-rank test revealed a statistically significant difference in ${\text{CNR}}_{mean}$ (W = 1330, *p* = 0.0039, n = 90) but no significant difference for ${\text{CNR}}_{max}$(W = 1960, *p* = 0.7248, n = 90).

This study revealed that DDMC increases the contrast of the spheres, as well as the background noise. A sub-analysis shows that the background noise increases with 15%, 61% and 37% for respectively TBR 2, 5 and 10.5, hence a 36% average increase in DDMC-reconstructed images.

## Discussion

In this study, images corrected with DDMC were compared to images without motion correction to determine whether DDMC improves the detectability of lesions. Phantom data with different sphere sizes, amplitudes, and TBRs were used to analyze the effect of the motion correction technique on the RC and CNR values.

DDMC visibly reduced motion-induced blur in target spheres and $\text{RC}$ values increased with DDMC, particularly for smaller spheres, higher TBR and larger motion amplitudes. Compared to other studies, this study has analyzed both maximal pixel RC and CNR values and the mean values of these parameters in a defined VOI. Using the mean values can lead to different conclusions; for example, ${\text{RC}}_{\text{mean}}$ values for larger spheres with small amplitudes, e.g., 7 mm, did not consistently improve. This difference in mean and max values arises because the maximal pixel $\text{RC}$ reflects the highest recovery coefficient within the sphere, capturing localized improvements that may occur due to motion compensation. In contrast, the mean RC accounts for the entire VOI, averaging variations and potentially diluting localized enhancements. For larger spheres with small motion amplitudes, the overall effect of DDMC may be less pronounced, as motion artifacts are less, leading to less consistent improvements in the mean $\text{RC}$ compared to the maximal pixel $\text{RC}$. This result aligns with prior research reporting that motion correction can degrade image quality for small motion amplitudes [21].

The ${\text{RC}}_{mean}$ is sensitive to the chosen VOI, which is challenging to determine in a moving object. In this study, we have proposed a method to determine the VOI. However, due to motion, the effective size of the VOI in the image may deviate from the nominal size, making it challenging to fully validate the suggested approach’s accuracy. The rigid VOI placement method can underestimate activity in motion-blurred targets. The rigid VOI placement method may not accurately capture targets, particularly for small spheres with large amplitudes. This effect is not expected to affect the conclusion’s validity, as the results of the small spheres with large amplitude align with other work.

The comparison of ${RC}_{max}$ and ${CNR}_{max}$ across different TBR values reveals that RC values tend to increase more noticeably with higher TBRs. Ideally, a stronger influence of DDMC would have been observed at lower TBRs, where detectability can be more uncertain. In contrast, CNR values do not show a clear trend, suggesting that TBR has little effect on CNR. For lesions that already have a high CNR, detectability is unlikely to improve further—once a lesion is clearly visible, additional contrast offers limited benefit.

The results show that the use of DDMC significantly improves the RC values and ${\text{CNR}}_{mean}$, while having no significant effect on the ${\text{CNR}}_{max}$. This indicates that DDMC may enhance overall image consistency without altering peak contrast performance. Despite the statistically significant improvements in the RC values and ${\text{CNR}}_{mean}$ with the use of DDMC, the distribution of individual differences reveals variability. This suggests that the effect of DDMC may be dependent on target and amplitude size. This is supported in the results, where particularly for small targets and big amplitudes, DDMC enhances RC and CNR values.

The improvement of image quality and RC aligns with previous studies, where the ability of DDMC was demonstrated to improve image quality and quantifications such as SUV [4, 12, 14]. Our study extends this work by evaluating the algorithm’s effectiveness across TBRs, motion amplitudes, and varying target sphere sizes, investigating the effects on RC and CNR. However, unlike previous research [4, 12] we observed a trade-off between improved contrast (RC) and increased background noise (${\sigma }_{B}$), which limited the overall detectability (CNR) of lesions. This discrepancy may be due to differences in study design, movement, or reconstruction algorithms. Dias et al. [14] have earlier shown that the coefficient of variation in gated reconstructions with DDMC were higher than without, and have shown that using DDMC reconstructions did not increase the detection of extra lesions.

The most important finding was that DDMC enhances the CNR of small, moving lesions, but CNR is reduced for lesions with little to no movement. This may also affect the detectability of small lesions. The increased background noise causes the effect but has also been previously appointed to be caused by image smoothing introduced during the elastic registration at the end of the EMC [7]. Unlike previous research that suggests no additional diagnostic information is obtained using DDMC [14], the changes in CNR values found by this study show that the detectability indeed can increase for some nodules. This finding suggests that DDMC can best be used selectively, carefully reviewing corrected and uncorrected images.

### Limitations of the study and suggestions for future research

Previous studies have investigated the effect of motion on tumor volume [12]. A limitation of our study is that we used the known volume of the target spheres and therefore do not account for the blurred volume as it appears as a spread target on the PET/CT. Future research could explore the effective volume of the target as it exists both in the phantom and patients. This would enhance our understanding of the algorithm, particularly its response to movement and the impact of the DDMC algorithm on lesion volume evaluation.

Another important consideration is how alternative reconstruction settings—such as different numbers of iterations/subsets, varying filter widths, or non-TOF reconstruction—might interact with DDMC and impact RC and CNR. While we anticipate that the noise increase observed with DDMC would be similar across different scanners and configurations, this assumption requires further validation. Future studies should explore how these factors influence the interplay between DDMC and quantitative accuracy.

Furthermore, literature has shown that mismatches in CT and PET for areas affected by respiration can occur [4]. The current study utilized attenuation correction with free-breathing CT. In a preliminary investigation on the SUV with and without DDMC using static and free breathing CT, we did not observe any difference in SUV within the image noise. To study the best CT-breathing technique in combination with DDMC, more research is required. In future work, it would be interesting to analyze the effect of PET and CT mismatches on DDMC, especially in patient data where more complex respiratory movement is seen.

This work relied on phantom experiments. The phantom’s simplified motion of 1D translation does not fully replicate respiratory motion complexity, which includes 3D rotation and heartbeat effects [21]. This study used the non-dedicated Kinetec Spectra to model respiratory movement, which may not fully capture the complexity of respiratory patterns. However, using phantom data is valuable in showing that background noise increases. In other patient studies, lesion-free tissue in the lung or liver is considered the background [4, 12]; this can lead to a wrong assessment of the background activity. The differences in the computation of background noise can be another cause for inconsistencies in CNR results in several studies. In future research, we will employ a dedicated device to model respiratory movements, offering enhanced functionality and greater accuracy, which is currently under development. An explanation video of the functioning of this device can be found under supplementary materials.

While phantom conditions mimicked clinical setups, real thoraco-abdominal motion is more complex, encompassing anterior–posterior and medial–lateral displacement, baseline drift, irregular breathing patterns, and hysteresis effects. However, our static data also demonstrate an increase in background noise (${\sigma }_{B}$), and beyond a certain threshold—where small spheres and large motion amplitudes are involved—the negative influence of higher background noise is outweighed by the positive impact of DDMC. Therefore, incorporating more complex motion patterns would not fundamentally change the conclusions of this study. To gain a deeper understanding of the trade-off between amplitude and lesion size in the presence of complex patient motion, we recommend a future study using simulated irregular respiratory patterns and patient data.

A study may validate our findings in a clinical setting by analyzing patient data with and without DDMC. To ensure a clinically relevant evaluation, detectability will be assessed by clinical observers. Additionally, to objectively validate whether DDMC techniques improve detectability, future work should incorporate a model observer, such as channelized hotelling observer (CHO) analysis [22].

This study has shown that DDMC improves RC and CNR in small lesions with high amplitudes.

## Conclusion

The DDMC algorithm reduced image blur, increased contrast, and increased image noise. Due to the increase in the noise in the background, the effect of DDMC is less profound than the effect of DDMC on the contrast. The CNR will increase for small lesions affected by respiratory motion with a high amplitude, which may lead to the detectability of these lesions with low uptake. Future work is needed to ensure that detectability improves, preferably in a clinical and multi-observer setting. DDMC reconstructions are recommended in addition to non-DDMC reconstructions due to the decreased detectability of lesions unaffected by motion.

## Supplementary Information

Supplementary file1: Video 1 The NEMA/IEC body phantom while moving in cycle mode with an amplitude of 3 cm and speed of 0.75 cm/s.

Supplementary file2: Video 2 Explanation of functioning of respiratory movement. Code To find VOIs in NEMA phanthom as described in methods section: https://github.com/mwinte11/VOI_identification_IQ

## Data Availability

The datasets analysed during the current study are available from the corresponding author on reasonable request.
